# Non-lethal assessment of the reproductive status of broadnose sevengill sharks (*Notorynchus cepedianus*) to determine the significance of habitat use in coastal areas

**DOI:** 10.1093/conphys/cou013

**Published:** 2014-05-30

**Authors:** Cynthia A. Awruch, Susan M. Jones, Martin García Asorey, Adam Barnett

**Affiliations:** 1 School of Zoology, University of Tasmania, TAS 7001, Australia; 2 Centro Nacional Patagónico (CENPAT), Conicet, Puerto Madryn, Chubut 9120, Argentina; 3 School of Life and Environmental Sciences, Deakin University, VIC 3125, Australia; 4 Fisheries, Aquaculture and Coasts Centre, IMAS, University of Tasmania, TAS 7001, Australia; 5 Centre for Tropical Water & Aquatic Ecosystem Research (TropWATER), Estuary and Tidal Wetland Ecosystems Research Group, School of Marine and Tropical Biology, James Cook University, Townsville, QLD 4811, Australia; 6 Oceans IQ, PO Box 200, Clifton Beach, Cairns, QLD 4879, Australia

**Keywords:** Elasmobranchs, maturity, steroid hormones, viviparity

## Abstract

Identification of the importance of habitats that are frequently used by any species is essential to a complete understanding of the species' biology and to incorporate their ecological role into conservation and management programmes. In this context, the present study investigated whether Tasmanian coastal waters have any reproductive relevance for the broadnose sevengill shark (*Notorynchus cepedianus*). Although this species is a large coast-associated apex predator in these areas, there is a complete gap in understanding the role that these coastal systems could play in its reproduction. Reproductive hormones were used as a non-lethal method to address the reproductive biology of this species. Females seemed to have at least a bi-annual reproductive cycle, being pregnant for ∼1 year and spending at least 1 year non-pregnant, with the ovulatory cycle separated from gestation. Mature females were found to be ovulating, in the initial stages of pregnancy, resting or starting a new vitellogenic cycle. *Notorynchus cepedianus* did not use these coastal habitats for mating or as a pupping ground. Although the mating season was distinguished between September to April, only 22% of males showed mating scars during the peak of the mating period and no near-term pregnant females were observed. Thus, despite these coastal waters being an important foraging ground for this species, these areas did not have any reproductive relevance. In consequence, future management and conservation planning programmes need to identify whether there are other areas in Tasmania that play a critical role for reproductive purposes in this species. Finally, although previous studies have linked reproductive hormones with external examination of the gonads to validate the use of steroids as a non-lethal tool to address reproduction, the present study used this methodology without killing any animals. This has important implications for conservation programmes of threatened and endangered species worldwide where the methodology cannot be validated.

## Abstract (precis)

Understanding the reproductive role that the habitat plays on animal behaviour is essential for conservation programs. Non-lethal endocrine methods were used to obtain reproductive information on the sevengill shark Notorynchus cepedianus habiting Tasmanian coastal areas to understand the reproductive significance that these systems might play on this large apex predator.

## Introduction

Reproduction is one of the most important events in the life of any living organism, because the primary requirement for successful propagation of any species and their individuals is the ability to reproduce. An understanding of the overall process of reproduction requires knowledge of the reproductive strategies, expressed through reproductive cycles, which are regulated by a combination of physical and biological variables (Bromage *et al.*, 2001; Pankhurst and Porter, 2003). An understanding of the reproductive cycle is fundamental if species are to be managed appropriately, so that they reproduce to maintain appropriate population levels. For fishery species and non-target species affected by fisheries, knowledge of the spatial and temporal timing of reproduction can ensure that fishery activities are minimized during reproductive periods (Baum *et al.*, 2003). Thus, identification of the roles that habitats/locations play for different stages of the reproductive cycle (e.g. spawning, birth, mating, ovulation or development) is crucial (Nemeth, 2005; Ashe *et al.*, 2010; Barnett *et al.*, 2012).

Traditionally, spatial and temporal patterns in reproduction of sharks have been obtained by killing animals and examining the condition of their gonads. In recent years, however, alternatives to traditional lethal techniques have been employed for generating necessary data without killing sharks (Hammerschlag and Sulikowski, 2011). Measurement of the circulating concentrations of plasma steroid hormones, such as 17β-estradiol (E_2_), progesterone (P_4_) and testosterone (T), can be used as a non-lethal technique to evaluate events associated with reproductive cycles in a number of sharks (Manire *et al.*, 1995; Koob and Callard, 1999; Sulikowski *et al.*, 2007; Awruch *et al.*, 2008, 2009).

In viviparous elasmobranch females, E_2_ has been associated mainly with both hepatic vitellogenin synthesis, leading to follicular growth, and reproductive tract development (Koob and Callard, 1999; Gelsleichter and Evans, 2012; Awruch, 2013).

The roles that androgens play during the reproductive cycle of viviparous females are poorly understood. Androgens showed no distinct patterns among the species where circulating steroids were tracked during the entire length of the reproductive cycle. Testosterone appears to be the primary androgen during follicular development, tracking closely with fluctuations in circulating E_2_ during ovulatory cycles (Rasmussen and Murru, 1992; Manire *et al.*, 1995; Koob and Callard, 1999), suggesting that androgens are likely to serve as a precursor for E_2_ synthesis. It has also been suggested previously that T plays a role in stimulating copulatory behaviour, as this steroid increased with mating period in some viviparous species, such as *Negaprion brevirostris*, *Dasyatis sabina*, *Sphyrna tiburo* and *Urobatis halleri* (Rasmussen and Gruber, 1993; Manire *et al.*, 1995; Snelson *et al.*, 1997; Tricas *et al.*, 2000; Mull *et al.*, 2010).

The primary role of P_4_ is in suppressing the hepatic production of vitellogenin to stimulate the maturation of ovarian follicles that have completed vitellogenesis (Pankhurst, 2008; Prisco *et al.*, 2008; Mull *et al.*, 2010). Additionally, circulating P_4_ levels were also reported to remain elevated during ovulation and the initial stage of pregnancy in viviparous elasmobranchs (Manire *et al.*, 1995; Snelson *et al.*, 1997; Tricas *et al.*, 2000; Prisco *et al.*, 2008; Mull *et al.*, 2010).

The broadnose sevengill shark, *Notorynchus cepedianus* (Péron 1807), is a common nearshore coastal species that is widely distributed in temperate coastal regions around the world, with the exception of the North Atlantic Ocean. This species belongs to the family Hexanchidae (Last and Stevens, 2009; Barnett *et al.*, 2012) and exhibits a lecithotrophic viviparous reproductive mode (Musick and Ellis, 2005). Studies on the reproductive cycle of the hexanchid sharks are very limited. Of the four species within the family, reproductive data on *Hexanchus nakamurai*, *Hexanchus griseus* and *Heptranchias perlo* are sourced from rather sporadic observations, while *N. cepedianus* has been studied in more detail (see Barnett *et al.*, 2012 for review). The reproductive cycle of *N. cepedianus* has not been determined because, to date, females have never been sampled throughout the entire reproductive cycle and only a single female has been found carrying near-term embryos (Ebert, 1986). Nevertheless, Ebert (1986, 1989) hypothesized a 6–12 month ovarian cycle and suggested that the ovarian cycle does not run in parallel with the 1 year gestation cycle. On the basis of the ovarian and gestation cycles, the length of the reproductive cycle for *N. cepedianus* would be 18–24 months (Ebert, 1989; Lucifora *et al.*, 2005).

In Australia, *N. cepedianus* is a major component of the elasmobranch assemblage in the coastal areas of south-east Tasmania (Barnett *et al.*, 2010a, b; Barnett and Semmens, 2012). The large number of *N. cepedianus* in these coastal systems and significant seasonal site fidelity over summer indicate that these areas are important habitats for this species (Barnett *et al.*, 2010b, 2011). The absence of smaller total length classes (<100 cm) from the catches suggests that *N. cepedianus* are not using these coastal habitats as pupping or nursery areas (Barnett *et al.*, 2010b). To date, the seasonal use of these habitats has been attributed to feeding site fidelity, where *N. cepedianus* move into coastal systems following seasonally abundant prey (Barnett *et al.*, 2011; Awruch *et al.*, 2012; Barnett and Semmens, 2012). However, it remains unknown whether this species is also using the area for reproductive purposes (e.g. mating, ovulation). In this context, the primary aim of the present study was to understand the reproductive role that Tasmanian coastal systems play for *N. cepedianus* by using non-lethal endocrine parameters to examine the reproductive status of this species and thereafter by linking this new information with previous studies on habitat utilization. In addition, this is the first time that reproductive hormones levels are reported for this species, which is important because it has significant potential to be used as a non-lethal method for future studies on this species worldwide but, more significantly, on other shark species with a similar reproductive mode.

## Materials and methods

### Data collection

**Figure 1:**
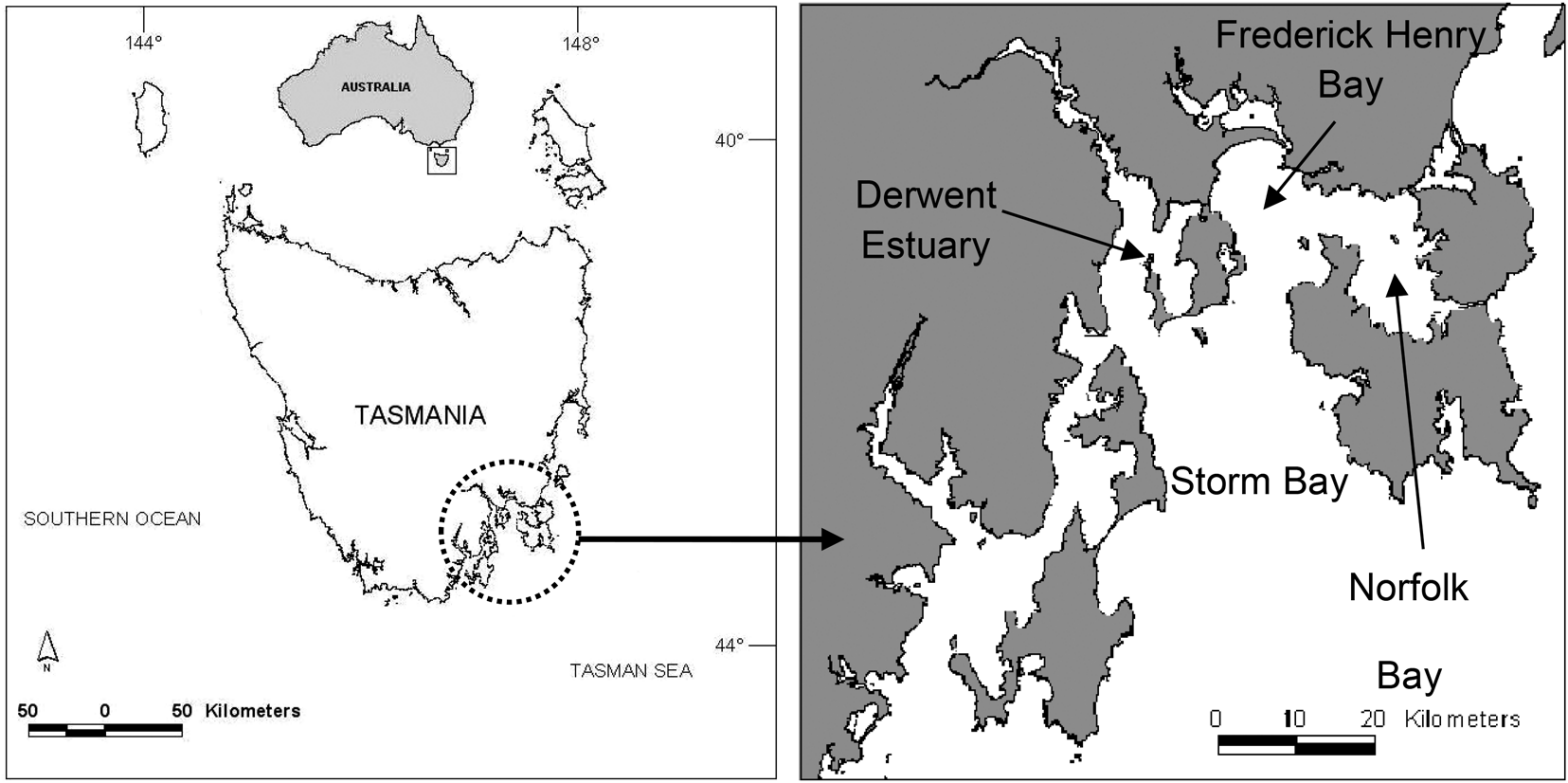
Study area of the south-east region of Tasmania showing sampling sites for *Notorynchus cepedianus*.

A total of 418 females and 159 males were used in this study. The majority of the samples (366 females and 129 males) were caught from Norfolk Bay and the Derwent Estuary, south-east Tasmania, Australia (Fig. 1) by fixed-site longline sampling between December 2006 and February 2009. In addition, 43 females and 23 males were taken from Derwent Estuary by opportunistic longline and rod and reel fishing, as well as nine females and seven males from Frederick Henry Bay by longline during the same time period. For detailed information on fishing methods see Barnett *et al.* (2010b).

Sex, total length (TL; in centimetres; measured over a straight line along the axis of the body from the tip of the snout to the posterior tip of the upper lobe of the caudal fin in its natural position), clasper calcification (determined by assessing the rigidity of the claspers by hand) and clasper length (CL; in centimetres; outer CL measured from the distal end of the metapterygium to the clasper tip and inner CL from anterior margin of the cloaca to the the posterior clasper tip) were recorded before sharks were released back into the water. The presence of mating scars was documented only from females caught by fixed longline sampling.

Seven females died on the line at the time of sampling. These females were dissected for visual examination of the reproductive tract, and the maximal follicular diameter (in millimetres), oviducal gland width (in millimetres), ovarian length (in centimetres), uterine width (in millimetres) and presence of embryos were recorded.

Blood samples (∼3 ml) from 69 females were collected by caudal venipuncture using pre-heparinized syringes fitted with 22 gauge needles. Although hormone analysis concentrated on females, blood samples were also collected in a subsample of 15 males. After extraction, blood samples were transferred to plastic vials and placed on ice for 3–6 h, followed by centrifugation for 5 min at 1000 × ***g***. The plasma was collected and stored at −15°C until being thawed for analysis.

### Steroid hormone measurement

Plasma levels of E_2_, T and P_4_ were measured in both sexes by radioimmunoassay. Plasma samples (200 µl) were extracted twice with ethyl acetate (1 ml), and 100 µl aliquots were transferred to assay tubes and evaporated before addition of an assay buffer. Testosterone, E_2_ and P_4_ antisera and [1,2,6,7-^3^H]E_2_, [1,2,6,7-^3^H]T and [1,2,6,7-^3^H]P_4_ were purchased from Sigma-Aldrich (Australia). The E_2_, T and P_4_ antisera were reconstituted by adding 5 ml of Tris buffer (pH 8, 0.1 m HCl), and 50 µl of [1,2,6,7-^3^H]E_2_, [1,2,6,7-^3^H]T and [1,2,6,7-^3^H]P_4_ were diluted in 5 ml of 100% ethanol and kept as stock for the assay. Duplicate standards (0–800 pg per tube authentic E_2_, T and P_4_ in ethanol) and sample extracts were evaporated, and 200 µl of the reconstituted antiserum, diluted 1:10 in assay buffer (containing 0.1% of gelatin and 0.01% of thiomersal), and 100 µl of the E_2_, T and P_4_ stock, diluted 1:9 in assay buffer, were added to each tube. Samples were placed in a batch at 37°C for an hour. Bound and free fractions were then separated using dextran-coated charcoal and aliquots of the supernatants counted in a Beckman LS 5801 liquid scintillation counter. All assays were validated by the evaluation of the slope of serially diluted extractions of plasma against the assay standards. The extraction efficiency was determined from recovery of ^3^H-labelled steroid added to 200 µl pooled aliquots of plasma and assay values were corrected accordingly. Extraction efficiency was 90% (E_2_), 92% (T) and 83% (P_4_). The detection limit for all assays was 0.02 ng (ml plasma)^−1^. Intra- and inter-assay variability was determined by including in each assay replicates of three levels of commercially available human control serum (CON4, CON5 and CON6 DPC). Inter-assay variability was 7% (E_2_), 10% (T) and 9% (P_4_) and intra-assay variability was <5% for all hormones.

### Data analysis

Females were not killed; therefore, using reproductive internal characteristics to address size at maturity was not possible. Reproductive hormone values obtained from the 69 blood samples were used to determine maturity. Levels of E_2_, T and P_4_ were plotted against TL to determine whether it was possible to distinguish any endocrine pattern linked with TL and, thus, use TL as the external characteristic to separate adult from juvenile females. For the reproductive cycle analysis, samples were combined into bi-monthly groups because of the low number of adult sharks present at some sampling times.

Clasper calcification was used to determine reproductive stages in males. Juveniles had uncalcified claspers, subadults partly calcified claspers and adults fully calcified claspers. To determine the size at which 50% of the male sharks mature, sharks were grouped into 10 cm length classes ranging from 110 to 250 cm. All fish that were not fully adults (e.g. subadults) were classified as juveniles. A logistic regression and standard error around the logistic model were calculated (Quinn and Keough, 2002).

In many elasmobranch males, an abrupt increase in clasper length near the length at maturity marks the onset of sexual maturity (Conrath, 2004; Francis and Duffy, 2005). Thus, outer and inner clasper lengths and clasper length index [CLI; obtained as (inner or outer CL/TL × 100)] were plotted against total length. To calculate the abrupt increase in clasper length, split linear regressions to the inner CLI data against TL were fitted. Split regressions consist of two straight-line segments fitted to different non-overlapping data ranges, which meet at a break-point (Kovác *et al.*, 1999; Francis and Duffy, 2005). Split regressions have the form:

$$\begin{array}{rl} & \mathrm{CLI}=a\times \mathrm{TL}+b\;\mathrm{for}\;\mathrm{TL}<p,\\ & \mathrm{CLI}=c\times \mathrm{TL}+d\;\mathrm{for}\;\mathrm{TL}\geq p, \end{array}$$

where *p* is the value of TL at which the slope and intercept of the relationship between CLI and TL changes, and with the continuity restriction that:

$$p=\frac{\left(d-b\right)}{\left(a-c\right)},$$

where *a* and *c* are the slope parameters for regression when TL < *p* and TL ≥ *p*, respectively, and *b* and *d* are the intercept parameters for the regression when TL < *p* and TL ≥ *p*, respectively. The split regression parameters were estimated by maximum likelihood, and confidence intervals for parameters were obtained by bootstrapping with a fixed-*x* resampling (Venables and Ripley, 2000; Fox, 2002).

All statistical analyses were done in R package 2.15.0 (R Development Core Team, 2012) at a critical probability level of 0.05.

## Results

### Size at maturity

**Females**

**Figure 2:**
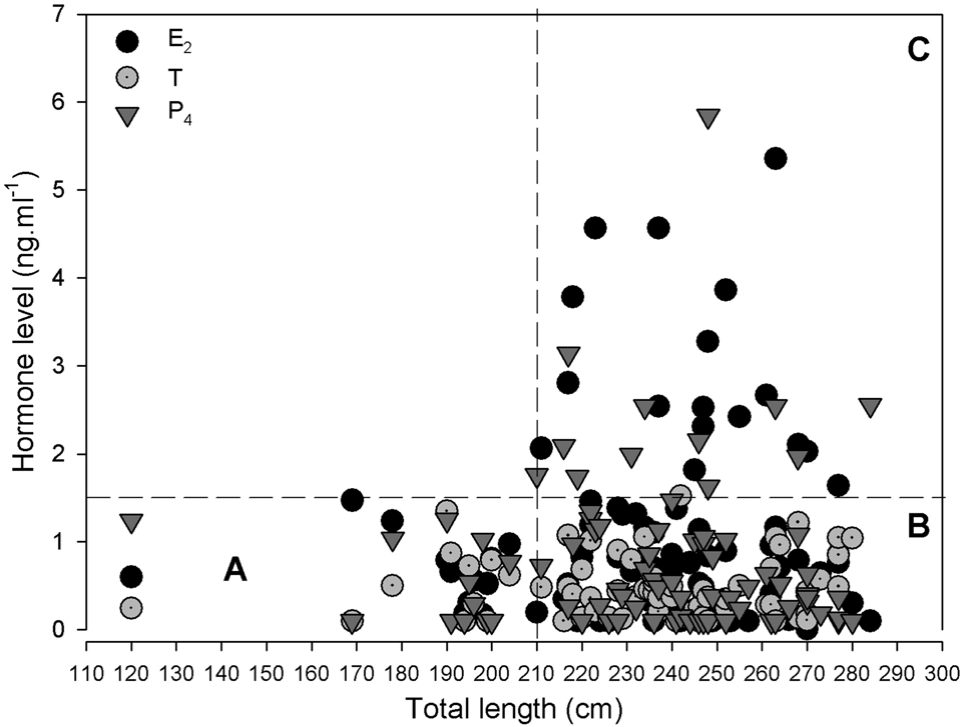
Relationship between hormone levels and total length in *N. cepedianus* females. Three different female groups were distinguished: A, B and C. *n* = 69. Abbreviations: E_2_, 17β-estradiol; P_4_, progesterone; T, testosterone.

The relationship between E_2_, T and P_4_ levels and TL for females showed a wide range of hormone levels at a similar TL, indicating different reproductive stages for a given TL. Looking into the relationship between hormone levels and TL, three groups can be distinguished visually, as follows (Fig. 2): females smaller than 210 cm TL that never reached hormone levels >1.5 ng ml^−1^ (group A) and females from 210 cm TL showing values higher and lower than 1.5 ng ml^−1^ (groups B and C). These results were probably indicating that group A females were sexually immature, while group B and C females were mature.

The difference between these last two groups (B and C) was probably a consequence of adult females in different stages of the reproductive cycle. The low values of females in group B, similar to juvenile females, were a result of adult females being either not sexually active (in a resting phase of the reproductive cycle) or starting a new vitellogenic cycle. Taking into account these results, 210 cm TL was chosen as the size at maturity for females. Thus, hormone levels for juvenile females ranged from 0.03 to 1.47 ng ml^−1^ for E_2_, from 0.03 to 1.35 ng ml^−1^ for T and from 0.03 to 1.25 ng ml^−1^ for P_4_; and for adults from 0.03 to 5.36, from 0.03 to 1.52 and from 0.03 to 5.84 ng ml^−1^ for E_2_, T and P_4_, respectively.

**Males**

**Figure 3:**
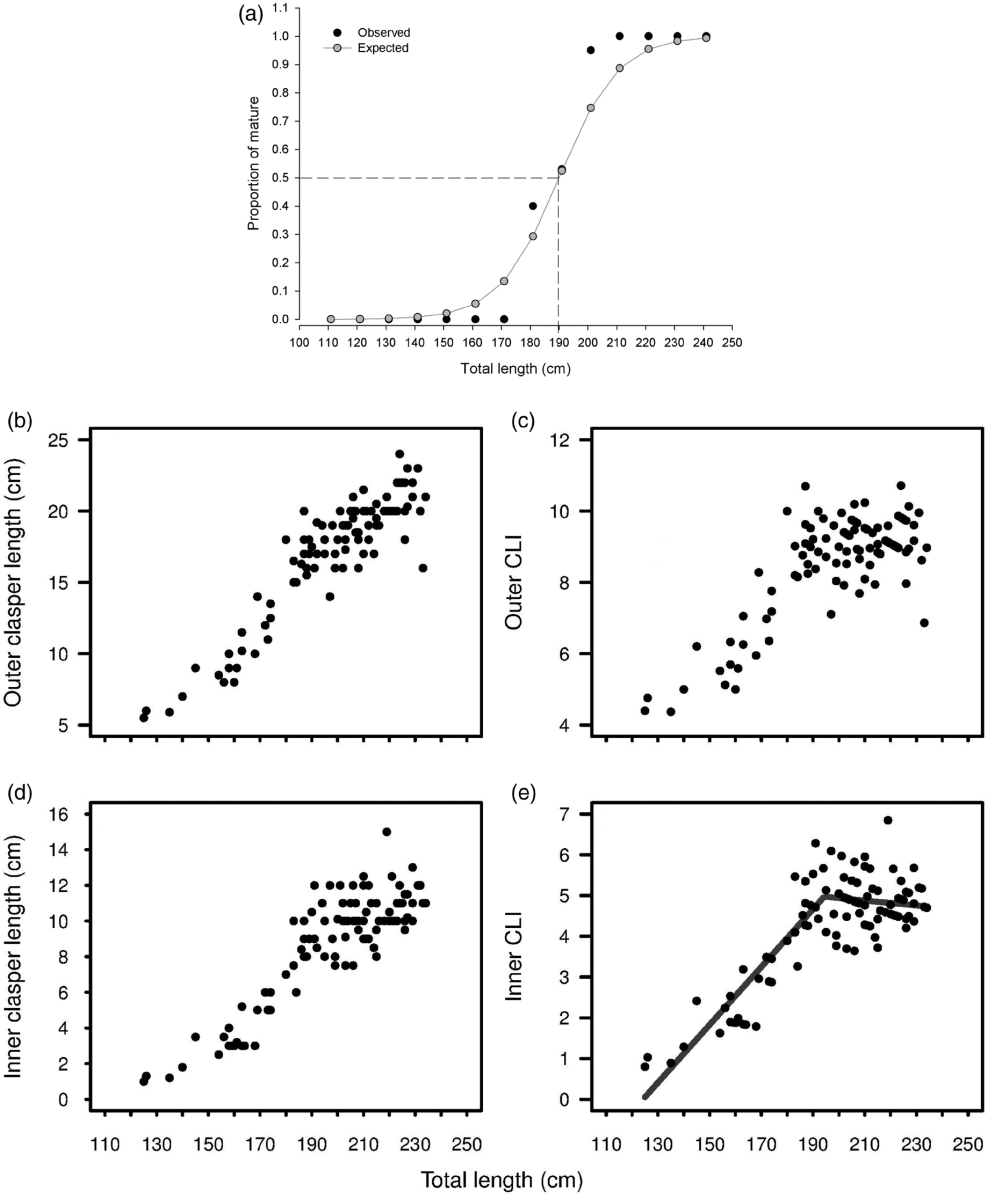
Maturation in *N. cepedianus* males. **(a)** Estimated maturity ogives. **(b)** Relationship between outer clasper and total length. **(c)** Relationship between inner clasper and total length. **(d)** Variation in outer clasper length index and total length. **(e)** Maturity estimation determined by split regressions between inner clasper length index and total length. *n* = 137. Abbreviation: CLI, clasper length index.

Males reached 50% size at maturity at 190–194 cm TL (Fig. 3). Males showed little overlap in length between immature and mature sharks; the maturity ogive estimated by the logistic regression was 190 cm (1.97 SE; Fig. 3a). The relationship between outer CL and TL was essentially linear and no apparent inflections were evident (Fig. 3b); thus, outer CL was not useful in estimating length at maturity. In contrast, an inflection point was observed when inner CL and outer and inner CLI were plotted against TL (Fig. 3c–e). Given that an abrupt increased in CL with TL was visually more inflected when inner CLI was plotted, this relationship was used to calculate the split regressions and the break-point between the regressions, being 194 cm (95% confidence interval 188.65–199.73 cm; Fig. 3e).

Male steroid levels (4 sexually immature and 11 sexually mature) showed differences between sexual stages for E_2_ (immature 0.52 ± 0.20 ng ml^−1^ and mature 1.53 ± 0.42 ng ml^−1^), T (immature 0.68 ± 0.20 ng ml^−1^ and mature 1.06 ± 0.12 ng ml^−1^) and P_4_ (immature 0.07 ± 0.04 ng ml^−1^ and mature 0.44 ± 0.14 ng ml^−1^).

### Reproductive cycle

**Figure 4:**
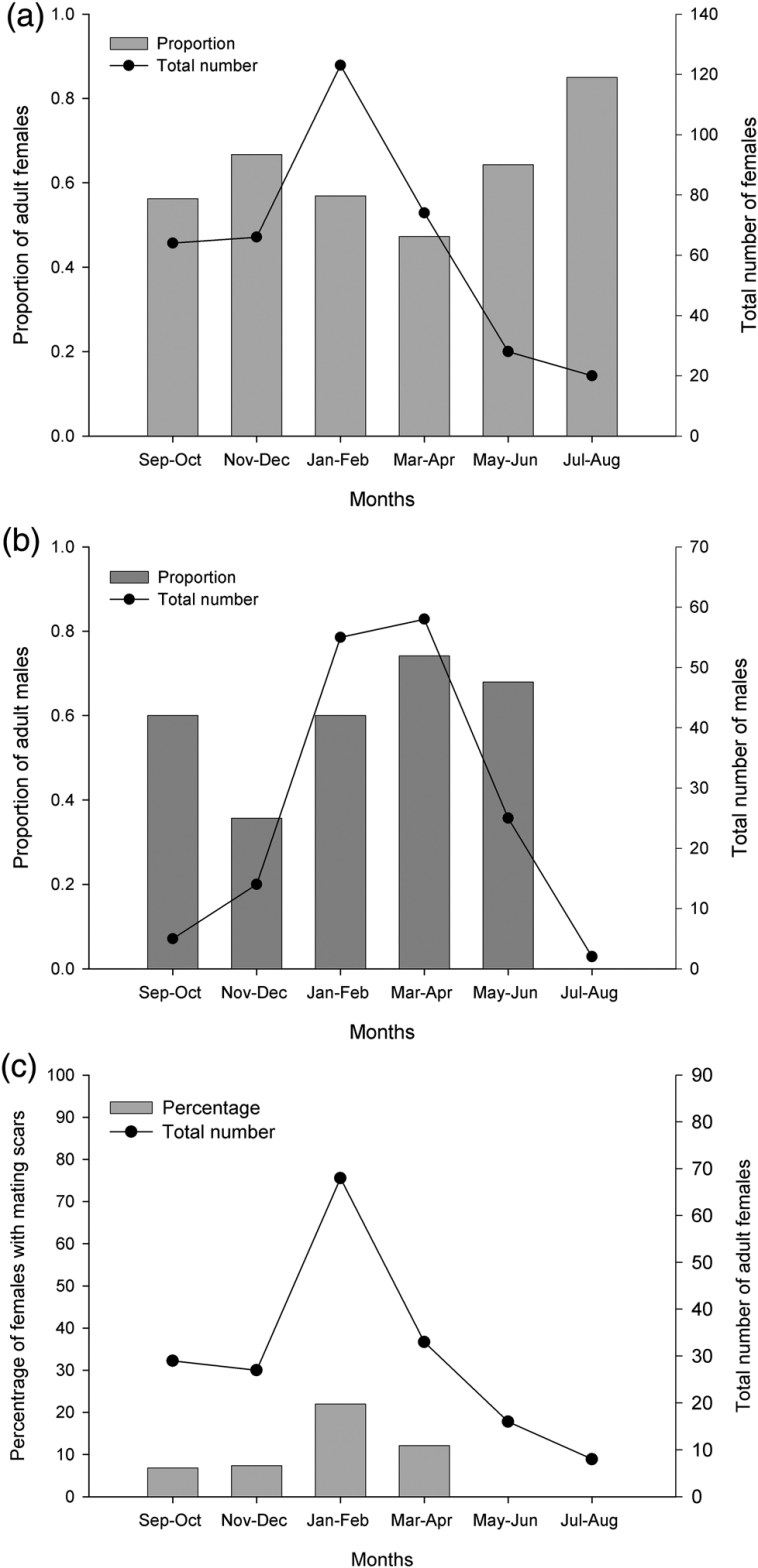
Bi-monthly variations of the proportion of *N. cepedianus* adult females **(a)** and males **(b)** throughout the study period. Proportion values are relative to the total number of females and males, respectively. **(c)** Percentage of females with mating scars.

The sex ratio throughout the complete study period was 2.4 female:1 male, with a proportion of 0.59 and 0.64 for adult females and males, respectively. When the data were split into 2 month periods, the proportion of adult females (relative to the total number of females) ranged from 0.47 to 0.87, with the majority of values falling between 0.47 and 0.67 (Fig. 4a) and the highest proportion (0.87) observed during the middle of the winter period, July–August (Fig. 4a). However, this concurred with the winter period having the lowest catch of total females (Fig. 4a). Bi-monthly proportions ranged from 0.36 to 0.74 in adult males. The proportion of adult males peaked during March–April, when occurrence of males in coastal areas is highest. No adult males were caught during July–August (Fig. 4b).

From the fixed longline sampling, females with mating scars started to appear in September (6% of total number of adult females), peaking during January–February (22%) before decreasing towards March–April (12%) to not being observed through the May–August period (Fig. 4c). All mating scars looked very fresh, except in December when one female looked to have older, well-healed mating scars. No juvenile females were seen with mating scars.

Of the seven dead females dissected for visual examination, two were juveniles and five adults. Females had a pair of ovaries, oviducts, oviducal glands and uteri. Both juveniles were caught in April (TL 100 and 200 cm), showing five to seven whitish follicles of 10 mm diameter, uterine width of 3–7 mm, oviducal gland width of 2–10 mm, and ovarian length of 10 cm. No detectable levels of P_4_ were found in any of these juvenile females; E_2_ levels ranged from 0.1 to 0.23 ng ml^−1^, and T levels were 0.27 ng ml^−1^ for both juvenile females. Blood samples were possible from only two of the adult females. Three adult females (two from January and one from February) showed long ovaries (∼40 cm long), with maximal follicular diameter between 63 and 70 mm and a second clutch of follicles of 10 mm diameter; each ovary contained between 32 and 37 follicles reaching the maximal size, with an oviducal gland width of 50–55 mm and uterine width of 40 mm. Corpora lutea or atresic follicles were seen in both females. One of the females from January (264 cm TL) had one oocyte (65 mm) moving into the uterus through the oviduct. The female caught in February (266 cm TL) showed fresh mating scars and had hormone levels of 2.43, 0.50 and 1.5 ng ml^−1^ for E_2_, T and P_4_, respectively. The other two adult females (both caught in January) had ovaries ∼18 cm long, with a main clutch of smaller (≤10 mm) white follicles and one or two whitish non-vitellogenic follicles between 15 and 17 mm; the ovaries were flaccid looking as if they were resting between reproductive events, with oviducal gland width between 24 and 35 mm and uterine width of 16 mm. One of these females (255 cm TL) showed hormone levels of 0.49, 0.45 and 0.24 ng ml^−1^ for E_2_, T and P_4_, respectively. None of these five females had visible embryos, indicating that the ovulatory cycle does not run in parallel with gestation.

**Figure 5:**
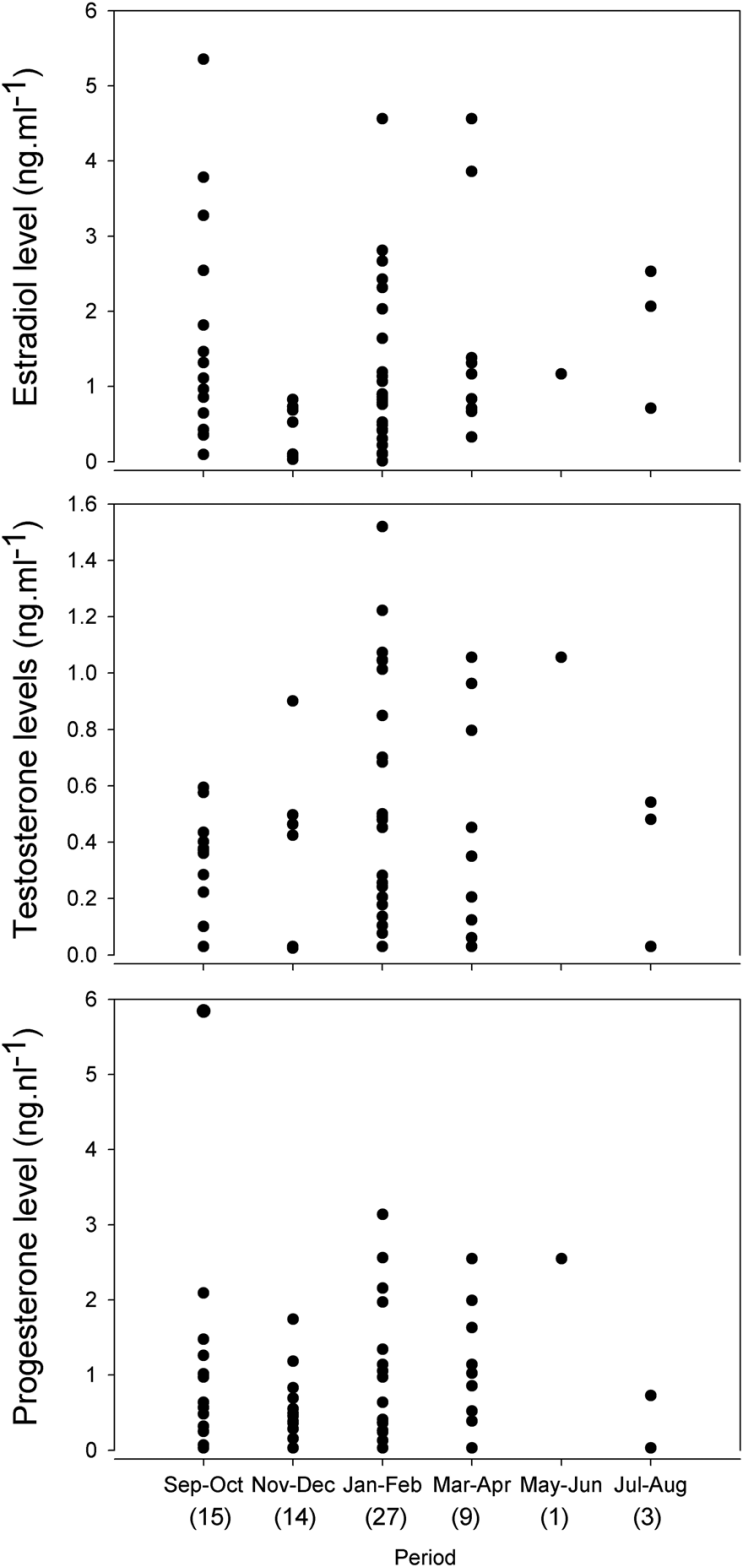
Bi-monthly variations of reproductive hormones in *N. cepedianus* adult females throughout the study period. Numbers in paretheses are sample sizes. Abbreviations: E_2_, 17β-estradiol; P_4_, progesterone; T, testosterone.

Data on adult female reproductive hormones showed a wide range of E_2_, T and P_4_ levels over the bi-monthly period throughout the year. Sample sizes from May to August were too small to reach any final conclusion; nevertheless, a trend in temporal periodicity can be distinguished (Fig. 5). The three steroid hormones showed a range of levels throughout the year, indicating that the reproductive cycle in *N. cepedianus* females is synchronous and multi-annual within the population (Fig. 5). The range of E_2_ levels throughout the year showed that from September to April some adult females are actively developing follicles within the ovary, while others are inactive (or not fully active), with steroid concentrations lower than 1.5 ng ml^−1^. These females were probably in a resting phase of the ovarian reproductive cycle or at the beginning of a new vitellogenic cycle (Fig. 5). Low levels of E_2_ were seen during November–December and May–June despite the low sample size. The reason for this particular pattern remains unknown, but it could be linked to the antagonistic effect between E_2_ and P_4_, which was more evident in May–June, when there were low E_2_ and high P_4_ levels.

Testosterone values were low (<1.6 ng ml^−1^) among all females (both juvenile and adult; Fig. 2). Throughout the year, for mature females the T levels tracked with fluctuations in circulating E_2_, peaking during January—February, which was when the majority of mating scars were visible, suggesting the possible role of androgens in stimulating copulatory behaviour (Fig. 5).

Circulating P_4_ titters reached the highest values during January–June, with a slight peak during January–February. In addition, one female showed a very high P_4_ value during September–October; however, it is important to note the 5.84 ng ml^−1^ P_4_ level could be an anomalous value. Given that mating scars were first noticed during September–October, with the majority being observed in January–February, these increases in P_4_ could be associated with the pre-ovulatory period, with P_4_ remaining elevated during the initial stage of pregnancy through May–June (Fig. 5).

### Reproduction by area

The two major areas of sampling, the Derwent Estuary and Norfolk Bay, were analysed separately. Nine females and seven males were excluded from this analysis, because they were caught in Frederick Henry Bay.

**Derwent Estuary**

**Figure 6:**
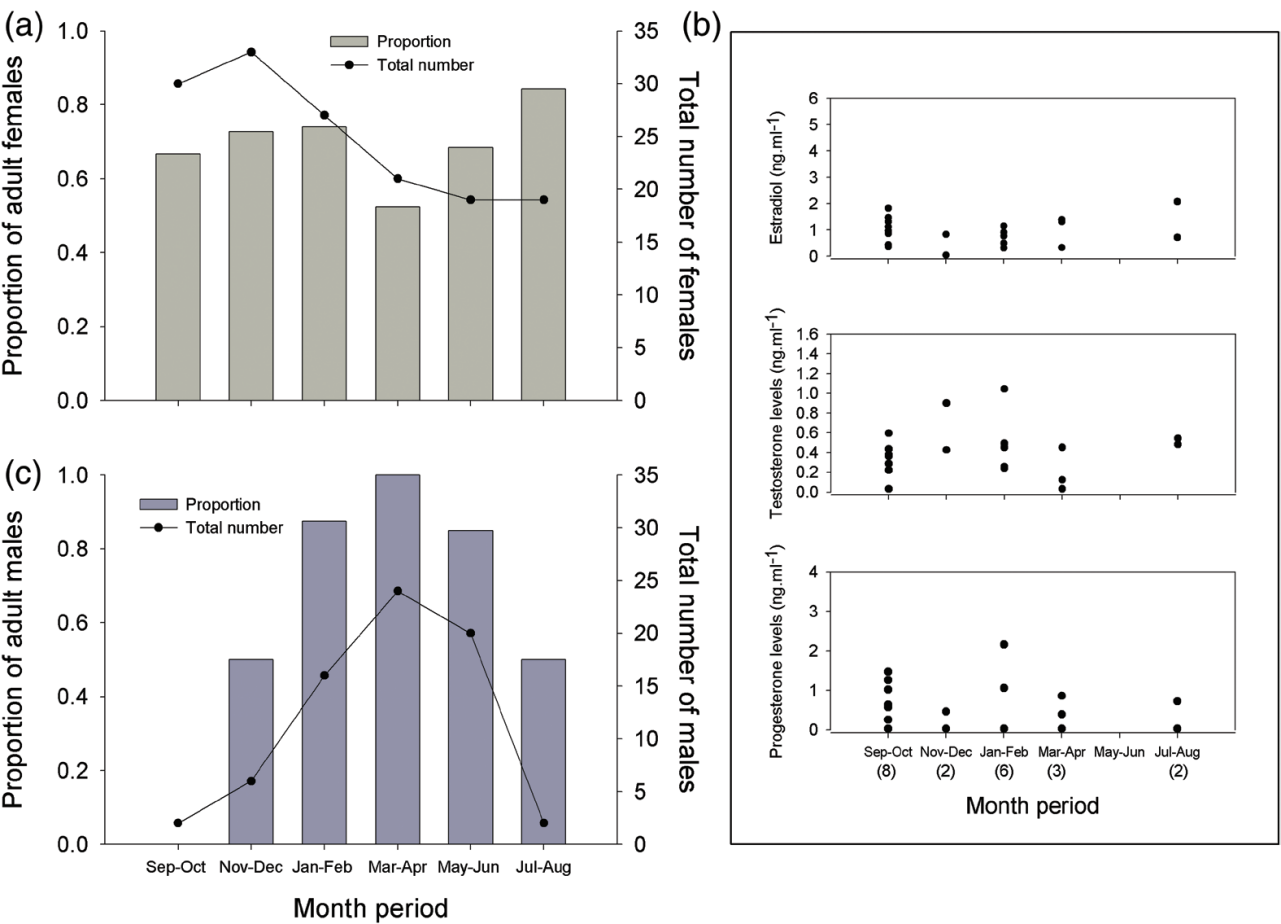
Bi-monthly variations of *N. cepedianus* from the Derwent Estuary. **(a)** Differences in the proportion of adult females. **(b)** Variations in 17β-estradiol (E_2_), testosterone (T) and progesterone (P_4_) levels in adult females. **(c)** Variations in the proportion of adult males.

The proportion of adult females caught in the Derwent area was 0.69 (total number of females, 149) and adult males 0.84 (total number of males, 70). At least 50% of all females present in the Derwent at any time of the year were adult, with proportions ranging closer to 0.7–0.8 for the majority of the months (Fig. 6a). Reproductive hormone data from adults showed that the majority of the E_2_, T and P_4_ values corresponded to the lower levels for each hormone, respectively (see Fig. 5). Hormone levels were below 2.06, 1.04 and 2.15 ng ml^−1^ for E_2_, T and P_4_, respectively (Fig. 6b), suggesting an important presence of adult females in the area, but not fully sexually active. Only two females (one in September–October and one in July–August) were probably undergoing vitellogenesis, with E_2_ values >1.5 ng ml^−1^, while two other females showing high P_4_ levels were probably ready to ovulate or pregnant during the September–February period (Fig. 6b). Of the total number of adult females sampled in the Derwent, 18% were observed displaying mating scars.

For males, the highest proportion of adults occurred between January and June, with only few males (of all sexual stages) caught from July to December (Fig. 6c).

**Norfolk Bay**

**Figure 7:**
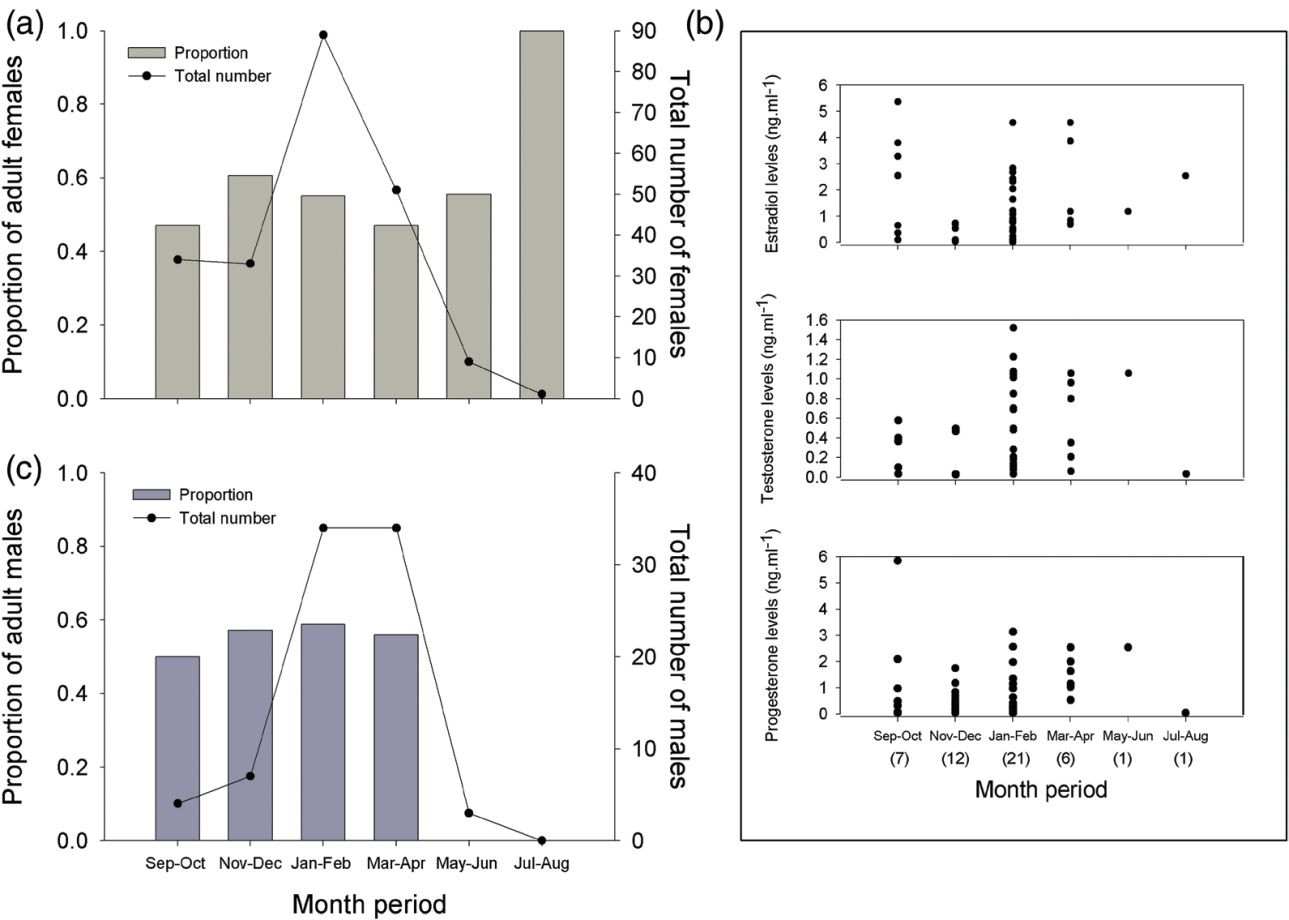
Bi-monthly variations of *N. cepedianus* from Norfolk Bay. **(a)** Differences in the proportion of adult females. **(b)** Variations in 17β-estradiol (E_2_), testosterone (T) and progesterone (P_4_) levels in adult females. **(c)** Variations in the proportion of adult males.

The proportion of adult females caught in Norfolk Bay was 0.53 (total number of females, 217) and adult males 0.52 (total number of males, 82). The proportion of adult females ranged between 0.4 to 1 from September to the July–August period (although only one female was caught during July–August; Fig. 7a), with 10% of these females carrying mating scars. Both sexually active and inactive females seemed to be in Norfolk Bay, as a wide range of the E_2_ (≤5.35 ng ml^−1^), T (≤1.52 ng ml^−1^) and P_4_ levels (≤5.84 ng ml^−1^) in each bi-monthly period (Fig. 7b). High levels of P_4_ could be an indication of ovulation and pregnancy maintenance. Given that few adult females between May and August were analysed for hormone levels, no definitive conclusions can be made for those periods. However, high levels of P_4_ can be seen for the only female caught in the May–June period, indicating that this female might possibly be pregnant. Likewise, the hormonal data from the female in the July–August period showed basal level of P_4_ and high levels of E_2_, indicating that this female was not pregnant and was undergoing an ovarian cycle (Fig. 7b).

The proportion of adult males was very similar, between 0.5 and 0.6 from September to April, with only three juvenile males caught between May and August (Fig. 7c).

## Discussion

In the last few years, several studies have investigated the habitat use of *N. cepedianus* (see Barnett *et al.*, 2012 for review). These studies concentrated mainly on movement patterns, diet and demography of this species in coastal environments. However, whether the use of coastal waters is associated with reproductive behaviour remains unknown. In this context, the present study provides novel information on *N. cepedianus* reproduction, linking the reproductive behaviour of this species with information previously reported on habitat utilization in coastal waters of Tasmania. This work employed non-lethal methodologies to obtain reproductive information on this species, which can be incorporated into elasmobranch management programmes that are already in place (DPIPWE, 2013).

### Size at maturity

Over the last decades, studies have increasingly suggested the use of non-lethal methods to address size at maturity in elasmobranch females. First, the presence or absence of a hymen has been used to indicate maturity; however, in some species the juvenile (or adolescent) females mate without being sexually mature (Pratt, 1979) and, additionally, the hymen could disintegrate naturally as the females grow (Pratt, 1979; Francis and Duffy, 2005). Second, ultrasonography allows the visualization of internal reproductive structures necessary to address sexual stages; however, to date, it has been unable to provide a very accurate and comprehensive assessment of maturity (Whittamore *et al.*, 2010). Third, reproductive hormones are indicators of maturation status in different elasmobranch species (Rasmussen and Murru, 1992; Rasmussen and Gruber, 1993; Gelsleichter *et al.*, 2002), where a single gonadal steroids or a combination of them have been proved recently to assess the onset of size at sexual maturity accurately (Sulikowski *et al.*, 2007; Awruch *et al.*, 2008).

The relationship between the reproductive hormones and TL showed 210 cm TL to be the size at maturity for *N. cepedianus* females. The marginal difference between T in juvenile and adult females showed that, for this species, T cannot be used to differentiate between the two sexual stages, and there was a need to measure only two hormones, E_2_ and P_4_, to separate juveniles from adults. The role that T plays in viviparous elasmobranchs remains unclear (Awruch, 2013) and, in this species, it is likely that T does not directly influence any physiological effects linked to maturity.

The size at maturity in *N. cepedianus* was slightly smaller than the 220–224 cm TL previously reported for *N. cepedianus* by visual examination of the reproductive organs of 17, 60 and 93 females from California (USA), South Africa and Patagonia (Argentina), respectively (Ebert, 1986, 1989, 1996; Lucifora *et al.*, 2005). However, 210 cm TL fell within the 95% confidence interval of the previously reported size at maturity (Ebert, 1996; Lucifora *et al.*, 2005). The acquisition of reproductive maturity is the attainment of fertility, when the increase in neuroendocrine secretions (gonadotrophin-releasing hormones) is essential for the activation and regulation of a process encompassing morphological, physiological and behavioural development (Ebling, 2005). Thus, as reproductive hormones regulate and trigger all processes of reproduction (Pankhurst, 2008), steroid changes that occur in *N. cepedianus* females at the onset of maturity may provide a more precise estimate of sexual development than morphological reproductive characteristics alone, as has been suggested previously for skate species (Sulikowski *et al.*, 2005, 2006).

Changes in relative size, calcification and development of the claspers are the most common external characteristics routinely used as tools for determining the stage of maturity in elasmobranch males (Callard *et al.*, 1988). It has been suggested that maturity assessments based on external characteristics alone could be imprecise (Pratt, 1979; Pratt and Tanaka, 1994), but in order to be sexually mature males must be able to produce viable sperm and have the capacity to deliver them. Although fully calcified claspers do not necessarily validate the ability to deliver viable sperm, the lack of completely developed claspers presumably prevents successful copulation. Thus, calcification of claspers is an external feature that can be readily assessed and is the most cost-effective method of identifying the size at sexual maturity of male sharks (Awruch *et al.*, 2008).

The combination of maturity stages, determined by clasper calcification, and an abrupt increase in inner CLI with TL were used as indicators of the onset of sexual maturity for *N. cepedianus* in the present study. These analyses showed a larger size at maturity, 191–194 cm (TL range 112–246 cm), than previously found in other regions. Size at maturity was reported to be 170 cm (TL range 153–217 cm), 150 cm (TL range 44–242 cm) and 160 cm (TL range ∼47–280 cm) for *N. cepedianus* from Patagonian, Californian and South African waters, respectively (Ebert, 1989, 1996; Lucifora *et al.*, 2005). In these cases, a slight increase in inner CL with TL was distinguished, and there was no (or little) overlap between juvenile and adult sharks. It is important to note that the sample sizes in the Patagonian and Californian studies were small (*n* = 43 for Lucifora *et al.*, 2005; and *n* = 60 for Ebert, 1989) and this could have affected the accuracy of the results. However, the sample size in South Africa was much higher (*n* = 172); therefore, the difference in size at maturity could be due sample sizes or a real difference in size at maturity for males from different regions. None of the previously reported values for *N. cepedianus* males fell within the confidence intervals obtained in the present study. In addition, although hormone analyses in this study concentrated on females, the preliminary steroid results in males showed differences between sexual stages supporting the morphological results.

### Reproductive cycle

As previously reported for *N. cepedianus* from different geographical regions (Ebert, 1989, 1996), females dissected in Tasmania carried a pair of ovaries, oviducts, oviducal glands and uteri. Females also showed two clutches of ovarian follicles, 10 and 60–70 mm in diameter, with oocytes found in oviducts at 65 mm diameter, suggesting that follicles reached ovulation size at 60–70 mm diameter. This maximal follicular diameter fell within the maximal follicular diameter reported by Ebert (1989, 1996) and Lucifora *et al.* (2005). However, different from *N. cepedianus* from California, South Africa and Argentina, fecundity seems to be much lower in specimens from Tasmania, with <40 follicles ready to be ovulated per cycle, in comparison with the higher values (60–100) for the specimens from the other regions.

Previous work on *N. cepedianus* in Tasmanian coastal waters showed a seasonal occurrence (spring and summer, Southern hemisphere) in the total number of individuals and a clear distribution biased towards females (Barnett *et al.*, 2010b). Likewise, the majority of mature females initially appeared during September–October, comprising ∼60% of the total female catch, and that number remained fairly constant until the end of March–April, when the proportion of mature females decreased to ∼40%. Sexually mature males followed females by peaking (to at least 60% of the total male catch) from January to April and decreasing to <30% from May.

The simultaneous presence of females ready to ovulate (some with fresh mating scars) and/or pregnant with females in a resting stage or starting a new ovulatory cycle indicated at least a 2 year reproductive cycle, where gestation is separate from the ovarian cycle. Likewise, looking into the hormonal data, a trend in temporal periodicity can be distinguished. The three reproductive hormones showed a range of levels throughout the sampling period, supporting the idea of a synchronous mode of reproduction within the population, where all females in the same reproductive stage go through the same reproductive events at the same time of the year.

The presence of mature females during September–April either ovulating or not fully sexually active (resting or at the beginning of a new vitellogenesis cycle) suggested that the ovarian cycle is at least 12 months long. Similar timing for yolk accumulation was suggested by Ebert (1989, 1996), and Daly *et al.* (2007) reported a possible 18–24 month period of follicular growth in *N cepedianus* in captivity. High levels of E_2_ found in sexually mature *N. cepedianus* are an indication of females undergoing follicular development. Looking at E_2_ levels, it could be inferred that follicular development occurs during the first year, reaching maximal size during spring–autumn (September–April), when follicular maturation and ovulation occur. This assumption is also supported by the higher levels of P_4_ that could be related to the onset of ovulation and early pregnancy, and the observation of a post-ovulatory follicle in female oviducts. In addition, high levels of E_2_ during the spring–autumn (September–April) period could also be associated with the protein secretions by the oviducal gland during the passage of the fertilized egg into the uterus, as was observed in other viviparous species, such as *Dasyatis sabina*, *Squalus acanthias* and *Sphyrna tiburo* (Tsang and Callard, 1987; Manire *et al.*, 1995; Tricas *et al.*, 2000; Callard *et al.*, 2005).

Although females are not mating in large numbers in Tasmanian coastal areas (see next subsection), the presence of mating scars in *N. cepedianus* during late spring (September) to autumn (April) was an indication of the mating season within the reproductive cycle for this species. High levels of P_4_ as an indicator of the onset of ovulation and, consequently, posterior sperm insemination together with high levels of T, which could be related to copulatory behaviour, were found during the same period. Nevertheless, taking into account that T levels could also increase as a result of other reproductive events, more evidence is necessary to support this idea. Additionally, mature males showing visible sperm around the claspers were found during January–April (Cynthia A. Awruch and Adam Barnett, personal observations) in coincidence with the higher proportion of mating scars.

A similar ovulation and mating period, in both duration and time of the year, were reported by Ebert (1989, 1996) for *N. cepedianus* inhabiting South African and Californian waters.

As mating appears to occur mainly between September and April, peaking in the January–February period, it is logical to assume that gestation starts in spring throughout autumn (Southern hemisphere), which was supported by the high levels of P_4_ found during this period, probably related to both ovulation and the initial stages of pregnancy (see review by Koob and Callard, 1999; Awruch, 2013). Pregnant females carrying near-term embryos (Ebert, 1986) showed a very noticeable increase in girth size (personal communication from Dr David Ebert, California Academy of Sciences). In addition, large females showing a distended abdomen have been regularly observed at an aggregation site in False Bay (South Australia; personal communication from Dr Alison Cock, South African Museum/University of Cape Town); it is hypothesized that these females may be in an advanced stage of pregnancy. As none of the females in the present study showed any enlargement of the abdominal region, it could be suggested that the possibly pregnant females found in these coastal areas are in the early stages of pregnancy.

Preliminary reports from recreational fishermen ( information collated by Jonah Yick) informed that neonatal *N. cepedianus* specimens are caught during spring–summer (Southern hemisphere) in Victoria (Australia), suggesting that the pupping season occurs during the warm seasons and that females are pregnant for a year. In concordance, a year gestation cycle, with pupping occurring during spring and summer, has been proposed for *N. cepedianus* from different regions (Ebert, 1989, 1996; Lucifora *et al.*, 2005). Similar to a previously report for *N. cepedianus* from California (Ebert, 1989), in the present study the presence of females with elongated ovaries (containing small, non-vitellogenic, whitish follicles) and very flaccid uteri was an indication that parturition had already occurred and that the new ovulatory cycle did not start immediately after parturition. However, the length of these resting periods remains unknown.

Thus, taking into consideration already published evidence and new information from this study, the length of the reproductive cycle in *N. cepedianus* could be suggested to be a 2 year cycle, in which females spend 1 year undergoing vitellogenesis and 1 year pregnant, with a short resting period before follicular development restarts, or a 3 year cycle, with similar timing for pregnancy but a longer follicular growth and/or resting period before a new ovulatory cycle resumes.

In summary, similar to viviparous elasmobranch species showing a seasonally punctuated cycle (Koob and Callard, 1999), *N. cepedianus* females are pregnant for approximately a full year, spending at least 1 year non-pregnant (which could be longer depending on the length of the vitellogenesis/resting period), with follicular development being separated from gestation.

### Ecological and conservation significance of coastal habitat use in southern Tasmania

Evidence thus far implies that these coastal areas are key seasonal foraging grounds (Barnett *et al.*, 2010a, 2011; Abrantes and Barnett, 2011; Barnett and Semmens, 2012). However, until the present study it was yet to be confirmed whether these coastal areas also had some reproductive function, e.g. for mating and/or developmental purposes.

Despite the finding that both Derwent Bay and Norfolk Bay had an important presence of adult females, the majority of the adult females in Derwent Bay were in the resting stages of the reproductive cycle or at the very beginning of the new ovulatory cycle, while in Norfolk Bay both resting and sexually active females (fully undergoing vitellogenesis or pregnant) were present. This separation in mainly non-active and active phases between the two locations was interesting, considering the strong site fidelity and low spatial overlap between *N. cepedianus* tagged in the Derwent Estuary and Norfolk Bay (Barnett *et al.*, 2011). It was expected that females would be found in all the different stages of reproduction at each site; however, if the sites have different reproductive relevance, it was then expected that animals would show more mixing (e.g. the resting phase in one site in 1 year and then moving to the other site the next year). The significance of this possible separation remains unclear. Further research, increasing the reproductive information and tracking studies, will be necessary to address whether these results are an artefact of small sample sizes in each area or real behaviour.

The lack of neonates or small juveniles (<100 cm) caught in coastal areas of Tasmania suggests that *N. cepedianus* do not use these areas as pupping or nursery grounds (Barnett *et al.*, 2010b). Based on the reproductive status of mature females, reproductive cycle information and mating scars being evident only on a low proportion of adult females captured during the mating season, it is suggested that these coastal areas in Tasmania have no significant/specific reproductive relevance. The most likely scenario is that *N. cepedianus* aggregations in these coastal areas are not specifically for mating and that any mating in these areas is probably opportunistic. Adult females in Tasmania were ready to ovulate, possibly pregnant, at the beginning of a new ovulatory cycle or they appeared to be in a reproductive resting phase, possibly related to a bi-annual reproductive cycle. Females approaching the ovulation phase could copulate in Tasmania or somewhere nearby, e.g. offshore Tasmania.

The absence of near-term pregnant females in such large seasonal aggregations containing both juvenile and adult *N. cepedianus* further raises the question of where near-term pregnant females and neonates are found in Australia. Recreational fisherman report catches of neonatal *N. cepedianus* in Port Philip Bay in Melbourne, Australia (∼700 km from the southern Tasmania coastal sites), but to date no scientific studies have been conducted in that area. There is also very little information on the habitat use of pregnant females in all regions of the world (Barnett *et al.*, 2012), which is a considerable gap in our understanding of the population ecology of *N. cepedianus.* Taking into account the previous stable isotope data indicating that Tasmanian offshore areas could be an important habitat for *N. cepedianus* (Abrantes and Barnett, 2011), it could be suggested that these habitats are refuges for females undergoing advanced stages of pregnancy.

In conclusion, the use of hormones to investigate the reproductive status of *N. cepedianus* in coastal protected areas clarified whether this species uses these areas for reproductive purposes, such as mating or pupping. Overall, although we can only speculate on where mating and pupping occur (Barnett *et al.*, 2010a, 2011; Abrantes and Barnett, 2011) and whether *N. cepedianus* have nursery areas at all, this study has ruled out the coastal areas of southern Tasmania as habitats with significant reproductive relevance. The information collated on this species in southern Tasmania strongly supports a previous hypothesis that these protected areas are important foraging grounds for *N. cepedianus* (Barnett *et al.*, 2010a, b; 2011; Barnett and Semmens, 2012). On average, *N. cepedianus* individuals spend 24% of their time in coastal protected areas of Tasmania (Barnett *et al.*, 2011), so they are vulnerable to exploitation for a significant amount of time. Given that there appears to be little reproductive significance to these habitats, the challenge for management and conservation planning is to determine whether there are habitats that are critical for reproductive purposes for this species, and where these habitats exist.
